# HIV-1 splicing is modulated by RNA structure

**DOI:** 10.1186/1742-4690-8-S2-P52

**Published:** 2011-10-03

**Authors:** Nancy Müller, Alex Harwig, Ben Berkhout, Atze T Das

**Affiliations:** 1 Laboratory of Experimental Virology, Department of Medical Microbiology, Center for Infection and Immunity Amsterdam (CINIMA), Academic Medical Center of the University of Amsterdam, Meibergdreef 15, 1105 AZ Amsterdam, The Netherlands

## Background

HIV-1 is a retrovirus with a ~9 kb RNA genome that contains 9 open reading frames and untranslated leader and tail regions. The full length RNA is used as genomic RNA that is packaged in the virion and as mRNA for translation of the Gag and Pol proteins. More than 40 differently spliced RNAs are produced that encode the other viral proteins. Splicing has to be strictly regulated in order to obtain the right balance between unspliced and spliced RNAs at the different phases of the viral life cycle. The 5'leader RNA encodes the major splice donor (SD) sequence that is used for the production of all spliced HIV-1 RNAs. This SD region can fold a stem-loop structure and the stability of this hairpin may be instrumental for the regulation of splicing by restricting the accessibility for the splicing machinery. In order to test this hypothesis, we varied the thermodynamic stability of the SD stem-loop through mutation and analysed the effects on virus replication and HIV-1 RNA splicing.

## Materials and methods

The SD stem-loop structure in the HIV-1 leader RNA region was stabilized or destabilized through mutation in the context of the HIV-1 molecular clone pLAI. The effect of the SD hairpin mutation on viral gene expression was measured upon transfection of 293T cells and virus replication was measured upon infection of SupT1 cells. The mutations were also introduced into an LTR-luciferase reporter plasmid that was designed to monitor SD splicing by causing reduced luciferase activity. Upon transfection of these constructs into 293T cells, the efficiency of splicing at the major SD site was analysed by luciferase activity assays and RNA analysis (RT-PCR).

## Results

Further stabilization of the SD stem-loop structure significantly reduced HIV-1 gene expression and replication and splicing was inhibited in the LTR-luciferase assay. SD hairpin destabilization did not cause major virus replication effects, but LTR-luciferase experiments revealed a modest upregulation of the splicing efficiency.

## Conclusions

The stability of the SD stem-loop structure modulates the frequency of splicing at the major SD site. These results suggest that the stability of this hairpin is fine-tuned to obtain the right balance between unspliced and spliced RNAs in HIV-infected cells.

